# The phenotypic and molecular characteristics of antimicrobial resistance of *Salmonella enterica subsp. enterica* serovar Typhimurium in Henan Province, China

**DOI:** 10.1186/s12879-020-05203-3

**Published:** 2020-07-15

**Authors:** Nian Dong, Yongrui Li, Jiayong Zhao, Hui Ma, Jinyan Wang, Beibei Liang, Xinying Du, Fuli Wu, Shengli Xia, Xiaoxia Yang, Hongbo Liu, Chaojie Yang, Shaofu Qiu, Hongbin Song, Leili Jia, Yan Li, Yansong Sun

**Affiliations:** 1 Chinese PLA Center for Disease Control and Prevention, 20th Dongda Street, Beijing, 100071 Fengtai District China; 2 Xingcheng Special Service Recuperation Center of PLA Strategic Support Force, 210th Xinghai South Road, Xingcheng, 125100 China; 3 Luoyang No.1 Hospital of TCM, 7th Jiudu Road, Luoyang, 471000 China; 4 The Key laboratory of Pharmacology and Molecular Biology, Medical College, Henan University of Science and Technology, 263th Kaiyuan Street, Luolong District, Luoyang, 471023 China; 5 Institute for Infectious Disease Control and Prevention, Henan Center for Disease Control and Prevention, Zhengzhou, China; 6 The Health Bureau of Logistical Support Department, Central Military Commission of China, 22 Fuxing Road, Beijing, 100036 Haidian District China; 7 State Key Laboratory of Pathogen and Biosecurity, Beijing Institute of Microbiology and Epidemiology, 20 Dongda Street, Beijing, 100071 Fengtai District China

**Keywords:** *S.* Typhimurium, Antibiotic resistance, Multidrug-resistant, ESBL, PMQR, Multilocus sequence typing

## Abstract

**Background:**

*Salmonella enterica subsp. enterica* serovar Typhimurium infections continue to be a significant public health threat worldwide. The aim of this study was to investigate antibiotic resistance among 147 *S.* Typhimurium isolates collected from patients in Henan, China from 2006 to 2015.

**Methods:**

147 *S.* Typhimurium isolates were collected from March 2006 to November 2015 in Henan Province, China. Antimicrobial susceptibility testing was performed, and the resistant genes of ciprofloxacin, cephalosporins (ceftriaxone and cefoxitin) and azithromycin were detected and sequenced. Clonal relationships were assessed by multilocus sequence typing (MLST) and pulsed-field gel electrophoresis (PFGE).

**Results:**

Of the 147 isolates, 91.1% were multidrug resistant (MDR), with 4.1% being resistant to all antibiotic classes tested. Of concern, 13 MDR isolates were co-resistant to the first-line treatments cephalosporins and ciprofloxacin, while three were also resistant to azithromycin. Seven PFGE patterns were identified among the 13 isolates. All of the isolates could be assigned to one of four main groups, with a similarity value of 89%. MLST assigned the 147 isolates into five STs, including two dominant STs (ST19 and ST34). Of the 43 ciprofloxacin-resistant isolates, 39 carried double *gyrA* mutations (Ser83Phe, Asp87Asn/Tyr/Gly) and a single *parC* (Ser80Arg) mutation, including 1 isolate with four mutations (*gyrA*: Ser83Phe, Asp87Gly; *parC*: Ser80Arg; *parE*: Ser458Pro). In addition, 12 isolates not only carried mutations in *gyrA* and *parC* but also had at least one plasmid-mediated quinolone resistance (PMQR) gene. Among the 32 cephalosporin-resistant isolates, the most common extended-spectrum β-lactamase (ESBL) gene was *bla*_OXA-1_, followed by *bla*_CTX-M_, *bla*_TEM-1_, and *bla*_CMY-2_. Moreover, the *mphA* gene was identified in 5 of the 15 azithromycin-resistant isolates. Four MDR isolates contained ESBL and PMQR genes, and one of them also carried *mphA* in addition.

**Conclusion:**

The high level of antibiotic resistance observed in *S.* Typhimurium poses a great danger to public health, so continuous surveillance of changes in antibiotic resistance is necessary.

## Background

*Salmonella enterica subsp. enterica* serovar Typhimurium is an important causative agent of human gastroenteritis and bacteremia in many countries. Worldwide, there are 93.8 million cases of human gastroenteritis due to *Salmonella* infection annually, associated with a death toll as high as 150,000 [1]. Over 2700 serovars have been identified using the White-Kauffmann-Le Minor scheme [1, 2], of which, *S.* Typhimurium is the most commonly associated with human and animal disease globally. *S.* Typhimurium is also the most prevalent serovars causing invasive Nontyphoidal *Salmonella* infections (iNTS), among iNTS cases, 63.7% occurred in children < 5 years of age globally [3]. Moreover, *S.* Typhimurium is the second most prevalent serotype in China [4].

Following the emergence of antimicrobial-resistant *Salmonella*, and in particular, the increasing prevalence of an epidemic multidrug-resistant (MDR) *S.* Typhimurium strain definitive phage type 104 (DT104) first observed during the 1990s [4], the antimicrobial resistance of *Salmonella* has become a matter of concern worldwide. Therefore, the appropriate selection of antimicrobial drugs in treating *Salmonella* infections is necessary. As previously described, cephalosporins (CEP) and ciprofloxacin (CIP) are first-line treatment agents for such infections [5], and azithromycin (AZI) has recently been approved by the US Food and Drug Administration as an additional therapy [6]. *Salmonella* isolates with reduced susceptibility to fluoroquinolones have been frequently reported in several countries, such as Kuwait, the United Arab Emirates, France [7–9]. Moreover, strains resistant to third-generation CEP have been described [10]. Given that these drugs are also first-line treatments for *Salmonella* infections in children, for whom fluoroquinolones are contraindicated, this represents a troubling development [10, 11]. Furthermore, a study of *Salmonella* in Cambodia revealed high rates of decreased susceptibility to CIP and AZI [12]. Worryingly, a recent report also identified a number of strains with concurrent resistance to CEP, CIP, and AZI in China [13].

Within China, significant regional differences in bacterial antibiotic resistance profiles have been demonstrated [14]. Henan is the most populous province of Central China and a region of substantial labor productivity. High levels of migration are expected to accelerate the spread of drug-resistant bacteria, exacerbating the threat to public health. Therefore, the objectives of this study were to determine the antimicrobial resistance profiles and molecular epidemiological characteristics of *S.* Typhimurium present in Henan between 2006 and 2015. This investigation will assist in establishing a scientific basis for the prevention and control of intestinal infectious diseases and guide the selection of appropriate antimicrobials for treatment of *S.* Typhimurium infections.

## Methods

### Bacterial isolates and serotypes

From 7 March 2006 to 28 November 2015, *S.* Typhimurium isolates were obtained from human patients with diarrhea and clinically suspected *Salmonella* infections, following previously described procedures [15]. The strains were isolated from 15 sentinel hospitals located in 11 different geographic regions in Henan Province, which covers an area of 167,000 km^2^ and is one of the most populous province (more than 95 million people) in China. Five of the hospitals were located in rural areas and the remaining 10 in urban areas. These hospitals consisted of second- and third-grade hospitals, township hospitals and village clinics. Patients generally have fever, diarrhea, abdominal pain, vomiting, watery stool and other clinical symptoms. Fresh fecal specimens collected from diarrhea patients of all ages were inoculated into Carry-Blair medium and forwarded to the regional CDC laboratories within 4 h. The stool samples were enriched in selenite brilliant green broth (Becton Dickinson and Co., Sparks, MD, USA) for 16–18 h at 37 °C, followed by incubation on *Salmonella/Shigella* agar (Land Bridge, Beijing, China) overnight at 37 °C. The resulting black colonies were streaked onto CHROMagar *Salmonella* agar (CHROMagar, Paris, France) and kept for 16–18 h at 37 °C to confirm their identity. The resultant purple colonies were then subjected to triple sugar iron agar, motility indole urea agar, l-lysine decarboxylase, and β-galactosidase (*o*-nitrophenyl-β-d-galactopyranoside) tests. Subsequently, a presumptive *Salmonella* colony from each sample was stored in semisolid agar and submitted to our laboratory for further confirmation. Age, gender, geographic origin, date and hospitals were recorded as part of the standard information present on the laboratory request forms. All isolates were identified using API 20E strips (bioMérieux Vitek, Marcy-l’Etoile, France) and tests for O and H antigens by slide agglutination with hyperimmune sera (State Serum Institute, Copenhagen, Denmark). Isolates were assigned to serovars according to the Kauffman-White scheme [7].

### Antimicrobial susceptibility testing

The susceptibility of each isolate to the following 13 antimicrobial agents was evaluated: ceftriaxone (AXO), cefoxitin (FOX), ampicillin (AMP), amoxicillin/clavulanic acid, 2:1 ratio (AUG2), CIP, nalidixic acid (NAL), AZI, tetracycline (TET), chloramphenicol (CHL), trimethoprim/sulfamethoxazole (SXT), sulfisoxazole (FIS), gentamicin (GEN), and streptomycin (STR). MICs were determined by broth microdilution using a 96-well microtiter plate (Sensititre CMV3AGNF, Trek Diagnostic Systems; Thermo Fisher Scientific, Inc., West Sussex, UK), with results being interpreted according to the recommendations of the Clinical and Laboratory Standards Institute (CLSI) [16]. Isolates resistant to three or more classes of antimicrobial agents were defined as MDR. The ACSSuT MDR profile, which was the prevalent resistance pattern in *S.* Typhimurium, was defined as resistance to AMP, CHL, STR, FIS, and TET [17]. *Escherichia coli* ATCC 25922 was used in susceptibility tests as a quality control strain, as specified by the Clinical and Laboratory Standards Institute.

### Multilocus sequence typing (MLST)

MLST analysis of the *S.* Typhimurium isolates was conducted according to previously described protocols [18]. Seven housekeeping genes *(thrA*, *dnaN*, *aroC*, *purE*, *hisD*, *hemD*, and *sucA*) were amplified by PCR using *Ex Taq* DNA polymerase (TaKaRa, Dalian, China) and primers whose sequences were downloaded from the abovementioned MLST resource. The PCR cycling conditions were as follows: 95 °C for 5 min; 30 cycles of 95 °C for 30 s, 55 °C for 45 s, and 72 °C for 1 min; and 72 °C for 7 min. The amplicons were sequenced by BGI (BGI, Beijing, China) and the resulting sequence data were checked against the MLST database [19] to establish sequence types (STs). The eBURST analysis was used with multilocus data to define groups of closely related isolates and display the relatedness between very different multilocus genotypes as previously described [20].

### Pulsed-field gel electrophoresis (PFGE)

*S.* Typhimurium isolates co-resistant to CIP and CEP were analyzed by PFGE according to the PulseNet protocol for *Salmonella* [21]. *Salmonella* standard strain Braenderup H9812 was employed as the molecular weight marker [22]. Agarose plug slices were digested with *Xba*I (TaKaRa) at 37 °C for 3 h, and electrophoresis was performed under the following conditions: voltage, 6 V/cm; switch time, 2.16–63.8 s (linear ramping); electric field angle, 120°; electrophoresis time, 19 h; and buffer temperature, 14 °C. PFGE patterns were analyzed using BioNumerics software version 6.0 (Applied Maths, Sint-Martens-Latem, Belgium), employing the Dice correlation coefficient and unweighted pair group method with arithmetic mean, with tolerance set to 1.2%.

### PCR amplification and DNA sequencing

Quinolone resistance-determining regions of the DNA topoisomerase genes *gyrA*, *gyrB*, *parC*, and *parE* were amplified by PCR assays as previously described [23]. Presence of the plasmid-mediated quinolone resistance (PMQR) genes *aac(6′)-Ib-cr*, *qnrA*, *qnrB*, *qnrD*, and *qnrS* was also established by PCR [24]. Amplification of antibiotic-resistance genes of the *bla*_TEM_, *bla*_SHV_, *bla*_OXA_, and *bla*_CTX-M_ groups and *bla*_CMY_ groups were performed by PCR for all isolates resistant to CEP [8, 24, 25]. In addition, for the AZI-resistant isolates, presence of the macrolide-resistance genes *mphA*, *mphB*, *ermA*, *ermB*, *ereA*, *mefA*, and *msrA* were tested [26]. PCR products were fully sequenced by BGI, and all nucleotide sequences were analyzed by comparisons against corresponding sequences in GenBank.

### Statistical analysis

The chi-squared test and Fisher exact probability test were used for data analysis in SPSS version 17.0 (SPSS Inc., Chicago, IL, USA). *P-*values < 0.05 were considered to indicate statistical significance.

## Results

### *S.* Typhimurium isolates from patients in Henan, China, from 2006 to 2015

A total of 147 *S.* Typhimurium isolates were recovered from 15 hospitals in 11 different geographic regions of Henan Province from 2006 to 2015, and the strains were distributed every year in each region (Table S1). Of these, 103 (70.1%) were isolated from patients below 6 years of age, and 91 (61.9%) from those less than 2 years old. Nine (6.1%) isolates were retrieved from patients 7 to 18 years of age, 21 (14.3%) from those 19 to 59 years of age, and 12 (8.1%) from those more than 59 years old. Two isolates (1.4%) derived from individuals of unknown age.

### Antimicrobial susceptibility testing

**Fig. 1:**
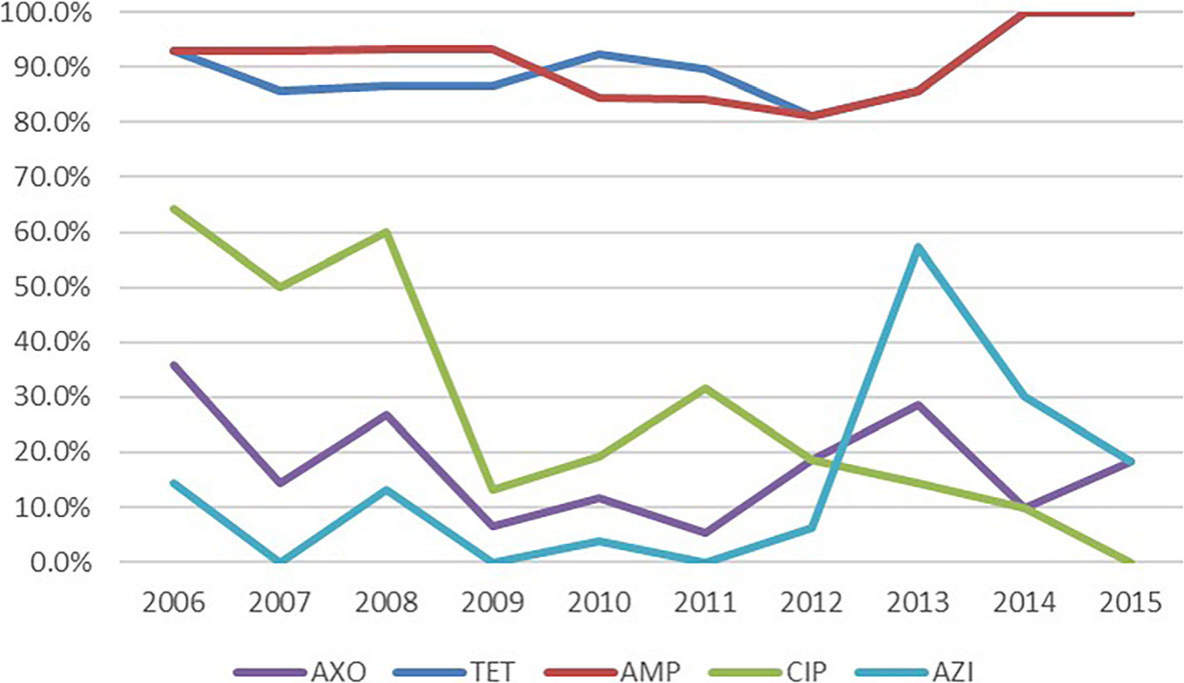
Trend of resistance rate of 5 common antibiotics in treatment between 2006 and 2015

The resistance patterns of the 147 *S.* Typhimurium isolates in response to the 13 antimicrobials tested are shown in Tables 1 and 2. There were 89% of the isolates which were resistant to AMP, TET or FIS, followed by NAL (115, 78.2%), SXT (114, 77.6%), CHL (113, 76.9%), GEN (102, 69.3%), STR (101, 68.7%), and AUG2 (97, 66%). Importantly, some isolates demonstrated strong resistance to at least one of four first-line treatment agents, namely, CIP (43, 29.2%), AXO (24, 16.3%), AZI (15, 10.2%), and FOX (9, 6.1%) (Table 1). All of the antimicrobial resistance levels of *S*. Typhimurium isolates in Henan were significantly higher than the level described in the 2014 National Antimicrobial Resistance Monitoring System report from the USA [27] (*P* < 0.05), and the resistance rate of the older generation of antibiotics (AUG2, NAL, CHL, SXT, GEN, STR) remained at a relatively high level, which showed a decline after 2011 (Fig. S1). Meanwhile, the resistance rate of two first-line treatment agents (FOX and AZI) remained at a relatively low level, which showed a rise after 2011. Particularly, the resistance rate of CIP began to decline significantly after 2008 from high (60%) to low (0%), but the resistance rate of AMP and TET remained at a high level (more than 80%) from 2006 to 2015, and the resistance rate of AXO was decreased from 35.7 to 18.2% (Fig. 1). Furthermore, 13 (8.8%) isolates were co-resistant to CIP and CEP, of which, three were also resistant to AZI.

**Table 1:** Antimicrobial susceptibility of 147 *S.* Typhimurium isolates collected from patients in Henan, China, between 2006 and 2015

Antimicrobial	No. isolates (%)			*NARMS Report n(%)	*P*-value
	Resistant	Intermediate	Susceptible		
β-Lactams					
Ceftriaxone	24 (16.3)	0 (0.0)	123 (83.7)	14 (5.3)	*P* < 0.05
Cefoxitin	9 (6.1)	0 (0.0)	138 (93.8)	14 (5.3)	*P* < 0.05
Ampicillin	132 (89.8)	0 (0.0)	15 (10.2)	52 (19.8)	*P* < 0.05
Amoxicillin/clavulanic acid, 2:1 ratio	97 (66.0)	0 (0.0)	50 (34.0)	14 (5.3)	*P* < 0.05
**Quinolones**					
Ciprofloxacin	43 (29.2)	0 (0.0)	104 (70.7)	1 (0.4)	*P* < 0.05
Nalidixic acid	115 (78.2)	0 (0.0)	32 (21.8)	7 (2.7)	*P* < 0.05
Macrolides					
Azithromycin	15 (10.2)	0 (0.0)	132 (89.8)	1 (0.4)	*P* < 0.05
Tetracyclines					
Tetracycline	132 (89.8)	0 (0.0)	15 (10.2)	59 (22.5)	*P* < 0.05
Amphenicols					
Chloramphenicol	113 (76.9)	2 (1.4)	32 (21.7)	42 (16.0)	*P* < 0.05
Sulfonamides and synergistic agents					
Trimethoprim/sulfamethoxazole	114 (77.6)	0 (0.0)	33 (22.4)	6 (2.3)	*P* < 0.05
Sulfisoxazole	132 (89.8)	0 (0.0)	15 (10.2)	66 (25.2)	*P* < 0.05
Aminoglycosides					
Gentamicin	102 (69.3)	1 (0.7)	44 (30.0)	8(3.1)	*P* < 0.05
Streptomycin	101 (68.7)	25 (17.0)	21 (14.3)	65 (24.8)	*P* < 0.05

* The antimicrobial resistant number and rate of 262 *S*. Typhimurium isolates were cited from the National Antimicrobial Resistance Monitoring System report from the USA in 2014 [27]

**Table 2:** Multidrug resistance patterns of *S.* Typhimurium isolates collected from patients in Henan, China, between 2006 and 2015

Multidrug resistance pattern	Number of isolates	Percentage	* NARMS Report(%)	*P*-value
≥ 3 antimicrobial classes	134	91.1	21.8	*P* < 0.05
≥ 4 antimicrobial classes	133	90.5	18.7	*P* < 0.05
≥ 5 antimicrobial classes	117	79.6	15.6	*P* < 0.05
≥ 6 antimicrobial classes	97	65.9	–	–
= 7 antimicrobial classes	6	4.1	–	–
ACSSuT	75	51.0	14.5	*P* < 0.05
ACSSuT+AZI	7	4.8	–	–
ACSSuT+CIP	31	21.1	–	–
ACSSuT+CEP	20	13.6	–	–
ACSSuT+CEP+CIP	10	6.8	–	–
ACSSuT+CEP+CIP + AZI	2	1.4	–	–
Sensitive to all	4	2.7	–	–

*CEP* cephalosporins, *CIP* ciprofloxacin, *AZI*, azithromycin

* The percentage of multidrug resistance patterns of *S*. Typhimurium isolates were cited from the National Antimicrobial Resistance Monitoring System report from the USA in 2014 [27]

**Fig. 2:**
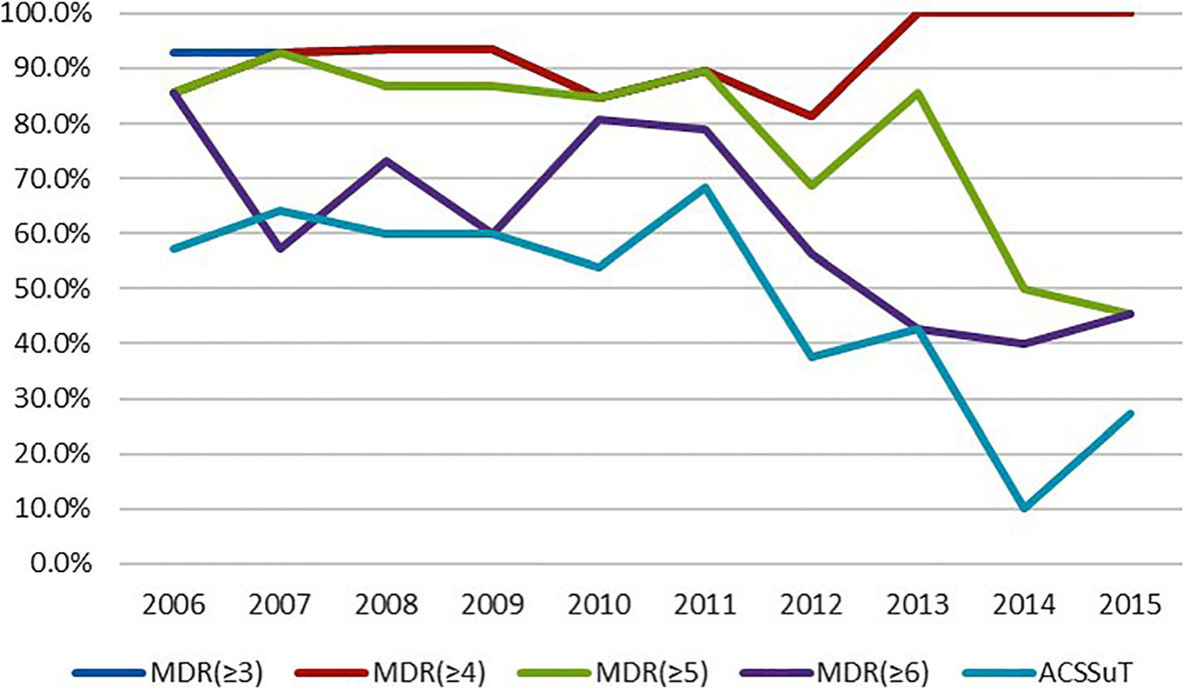
The change of multidrug resistance pattern of *S.* Typhimurium isolates collected from patients in Henan, China, between 2006 and 2015. MDR (≥3): ≥3 antimicrobial classes, MDR (≥4): ≥4 antimicrobial classes, MDR (≥5): ≥5 antimicrobial classes, MDR (≥6): ≥6 antimicrobial classes

In total, 134 (91.1%) isolates were MDR (Table 2). Of these, 133 (90.5%), 117 (79.6%), 97 (65.9%), and six (4.1%) were resistant to at least four classes of antimicrobials, five classes of antimicrobials, six classes of antimicrobials, and all classes of antimicrobials tested, respectively. Seventy-five (51.0%) MDR isolates exhibited the ACSSuT resistance pattern, of which, 41.3% were co-resistant to CIP, 26.6% were co-resistant to CEP, and 9.3% were co-resistant to AZI. Notably, two (1.4%) ACSSuT-type isolates were co-resistant to all three of these antimicrobials (Table 2). The MDR (≥3 and ≥ 4 antimicrobial classes) rate remained at a high level, while MDR (≥5 and ≥ 6 antimicrobial classes) and ACSSuT resistance pattern showed a decline over time (Fig. 2). But the rates of MDR (≥3, ≥4 and ≥ 5 antimicrobial classes) and ACSSuT were significantly higher than the rate described in the 2014 NARMS report [27] (*P* < 0.05) (Table 2).

### MLST and PFGE analyses

**Fig. 3:**
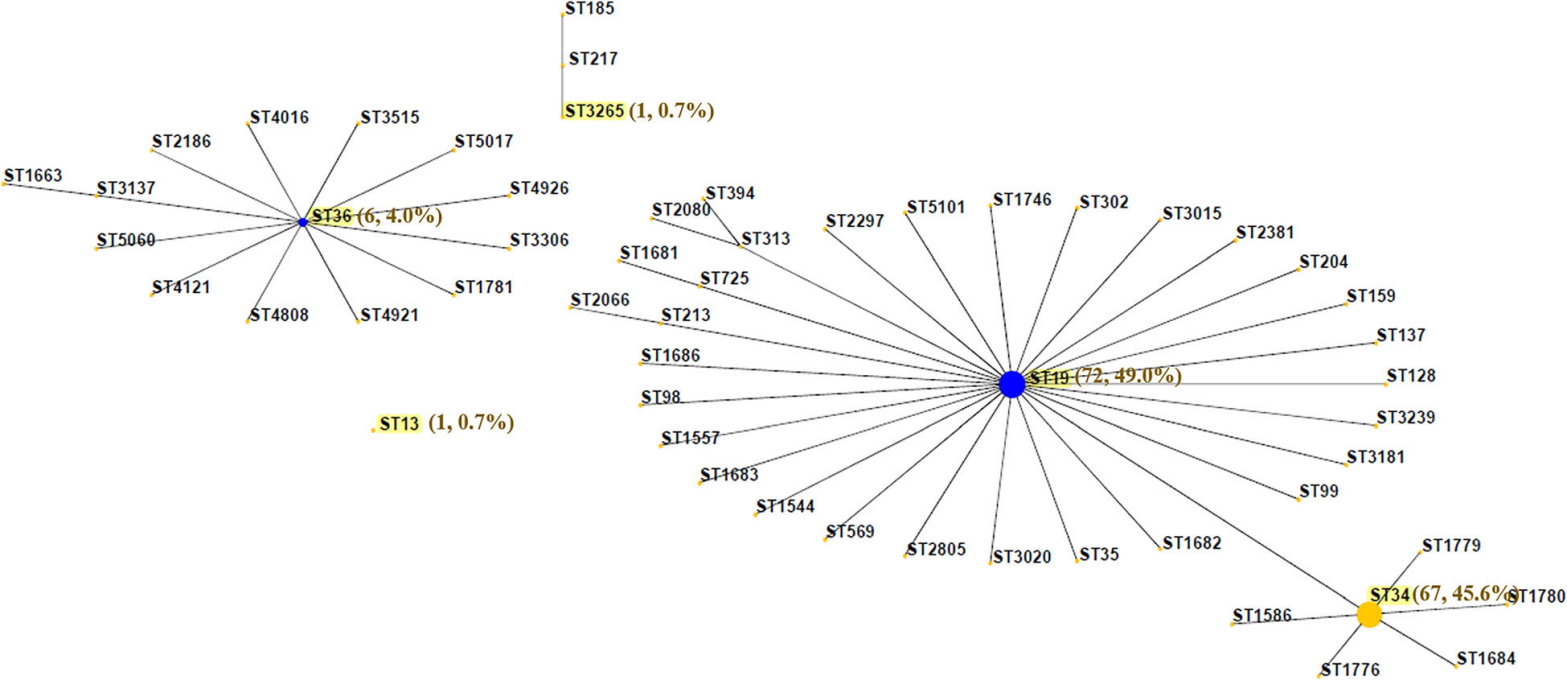
The eBURST diagram for sequence types (STs) among *S.* Typhimurium isolates. The eBURST diagram was generated with STs of 147 *S*. Typhimurium detected in this study and other STs in the MLST database

**Fig. 4:**
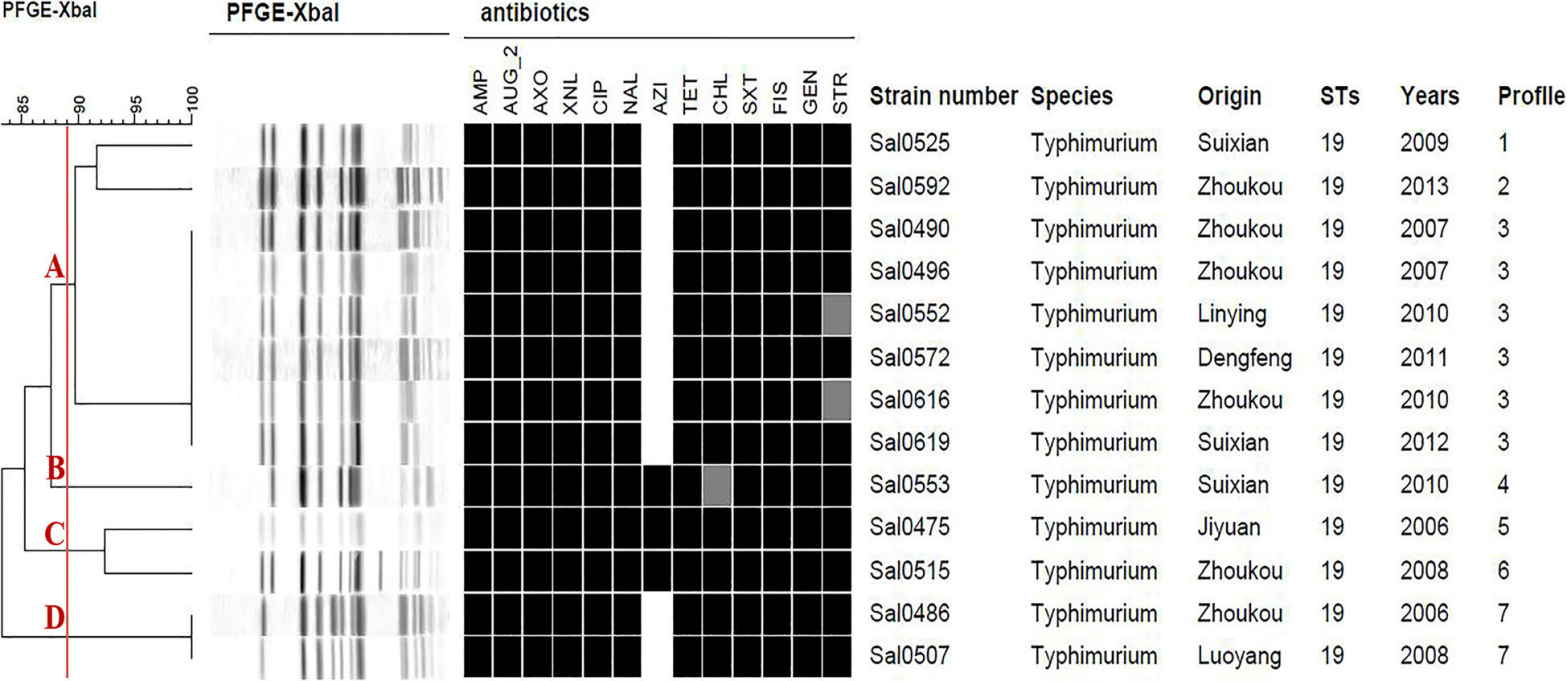
Pulsed-field gel electrophoresis (PFGE) dendrogram, antibiotic resistance profiles, and sequence types (STs) of clinical *S.* Typhimurium isolates resistant to both ciprofloxacin and cephalosporins. Strain number, year of isolation, place of origin, species, profile number and ST are listed after each PFGE profile. FOX, cefoxitin; AXO, ceftriaxone; AZI, azithromycin; CHL, chloramphenicol; TET, tetracycline; CIP, ciprofloxacin; GEN, gentamicin; NAL, nalidixic acid; FIS, sulfisoxazole; AMP, ampicillin; STR, streptomycin; AUG_2, amoxicillin/clavulanic acid, 2:1 ratio; SXT, trimethoprim/sulfamethoxazole. Black indicates “resistant,” grey “intermediate susceptibility,” and white “sensitive”

MLST assigned the 147 isolates into five STs as follows: ST19 (72, 49.0%), ST34 (67, 45.6%), ST36 (6, 4.0%), ST3265 (1, 0.7%), and ST13(1, 0.7%) (Fig. 3). The eBURST analysis showed there were four eBURST groups between the five STs, ST19 and ST34 belonged to one group, and ST36, ST3265 and ST13 belonged to another groups, respectively. ST19 and ST34 are single locus variants (SLVs) in which one of the seven MLST loci has been altered, due to different sequence values of *dnaN*. PFGE was performed to determine the genetic relatedness of those isolates co-resistant to CIP and CEP. Seven PFGE patterns (Profile 1–Profile 7) were identified among the 13 isolates. All of the isolates could be assigned to one of four main groups (A–D), with a similarity value of 89%. PFGE patterns revealed both diversity and the predominance of certain profiles. Eight isolates belonged to group A, in which three profiles were observed and Profile 3 was the main pattern type including six isolates. Group C and group D contained two isolates each, while only one isolate was present in group B (Fig. 4).

### PCR amplification and DNA sequencing

Mechanisms of resistance in the 43 CIP-resistant *S.* Typhimurium isolates were investigated. All of these isolates were found to have *gyrA* mutation and 39 (90.7%) of them carrying two mutations (Ser83Phe and Asp87Asn/Tyr/Gly), and four (9.3%) carrying only one (Asp87Asn or Asp87Tyr). Thirty-nine isolates (90.7%) had the *parC* Ser80Arg mutation. In addition, double *gyrA* mutations together with a single *parC* (Ser80Arg) mutation were detected in 38 (88.4%) of the 43 isolates. None carried a *gyrB* mutation; however, a *parE* mutation (Ser458Pro) was identified in one isolate, which interestingly also carried two *gyrA* mutations (Ser83Phe and Asp87Gly) and a single *parC* (Ser80Arg) mutation. Furthermore, two PMQR determinants were detected: *qnrS1* (11 isolates, 25.6%) and *aac(6′)-Ib-cr* (seven isolates, 16.3%). Among the four isolates with a single *gyrA* mutation (Asp87Tyr or Asp87Asn), two had the *aac(6′)-Ib-cr* gene, one had the *qnrS* gene and one did not have either. None of the isolates carried *qnrA*, *qnrB*, or *qnrD*, and 28 (65.1%) of 43 isolates didn’t carry any PMQR determinants tested.

The 32 *S.* Typhimurium isolates found to be resistant to CEP antibiotics all carried β-lactamase gene and 16 (50%) had at least two ESBL genes. PCR amplification and sequencing showed that 28 (87.5%) isolates were positive for *bla*_OXA-1_, 13 (40.6%) for *bla*_CTX-M_, nine (28.1%) for *bla*_TEM-1_, and two (6.3%) for *bla*_CMY-2_. All 32 isolates were negative for *bla*_SHV_. Furthermore, sequencing revealed the presence of *bla*_CTX-M-14_, *bla*_CTX-M-15_, and *bla*_CTX-M-55_ in six, two, and five isolates, respectively.

PCR showed that only five (33.3%) of the 15 azithromycin-resistant isolates were positive for *mphA*, and none of them carried *mphB*, *ermA*, *ermB*, *ereA*, *mefA*, or *msrA*.

Among the 13 isolates resistant to both CIP and CEP, 8 harbored ESBL and carried two mutations in *gyrA* and one in *parC*, one harbored ESBL and carried one mutation in *parC*, 4 harbored ESBL and PMQR genes (Table 3). Moreover, each of these four carried two mutations in *gyrA* and one in *parC*. Two isolates each harbored four types of antimicrobial-resistance gene: *qnrS*/*aac(6′)-Ib-cr*/*bla*_OXA_/*bla*_CTX_ (*n* = 1) and *aac(6′)-Ib-cr*/*bla*_OXA_/*bla*_CTX_/*bla*_TEM_ (*n* = 1). One isolate exhibited three antimicrobial-resistance gene types (*qnrS*/*bla*_CTX_/*bla*_OXA_), and one other was found to have two types (*qnrS*/*bla*_OXA_). In addition, 2 of the 13 isolates harbored the *mphA* gene.

**Table 3:** Antimicrobial resistance determinants of clinical *S.* Typhimurium isolates resistant to both ciprofloxacin and cephalosporins

Strain	Years	STs	QRDR mutations		PMQR genes		β-Lactamase genes and types				Macrolide resistance gene
			*gyrA*	*parC*	*qnrS*	*aac(6′)-Ib-cr*	*bla*_CTX-M_	*bla*_OXA_	*bla*_CMY-2_	*bla*_TEM-1_	*mphA*
Sal0475	2006	19	Ser83Phe, Asp87Asn	Ser80Arg	–	–	*bla*_CTX-55_	*bla*_OXA-1_	*bla*_CMY-2_	–	–
Sal0485	2006	19	Ser83Phe, Asp87Asn	Ser80Arg	–	–	*bla*_CTX-15_	*bla*_OXA-1_	–	–	–
Sal0490	2007	19	Ser83Phe, Asp87Asn	Ser80Arg	–	–	*bla*_CTX-55_	*bla*_OXA-1_	–	–	–
Sal0496	2007	19	Ser83Phe, Asp87Asn	Ser80Arg	–	–	*bla*_CTX-55_	*bla*_OXA-1_	–	–	–
Sal0507	2008	19	Ser83Phe, Asp87Asn	Ser80Arg	–	–	*bla*_CTX-15_	*bla*_OXA-1_	–	–	–
Sal0515	2008	19	Ser83Phe, Asp87Asn	Ser80Arg	–	*aac(6′)-Ib-cr*	*bla*_CTX-55_	*bla*_OXA-1_	–	*bla*_TEM-1_	*mphA*
Sal0525	2009	19	Ser83Phe, Asp87Asn	Ser80Arg	–	–	–	*bla*_OXA-1_	–	–	–
Sal0616	2010	19	Ser83Phe, Asp87Asn	Ser80Arg	–	–	*bla*_CTX-14_	*bla*_OXA-1_	–	–	–
Sal0552	2010	19	Ser83Phe, Asp87Asn	Ser80Arg	–	–	*bla*_CTX-14_	*bla*_OXA-1_	–	–	–
Sal0553	2010	19	Asp87Tyr	WT	–	–	–	*bla*_OXA-1_	–	–	*mphA*
Sal0572	2011	19	Ser83Phe, Asp87Asn	Ser80Arg	*qnrS1*	–	*bla*_CTX-55_	*bla*_OXA-1_	–	–	–
Sal0619	2012	19	Ser83Phe, Asp87Asn	Ser80Arg	*qnrS1*	*aac(6′)-Ib-cr*	*bla*_CTX-55_	*bla*_OXA-1_	–	–	–
Sal0592	2013	19	Ser83Phe, Asp87Asn	Ser80Arg	*qnrS1*	–	–	*bla*_OXA-1_	–	–	–

*QRDR* quinolone resistance-determining region, *PMQR* plasmid-mediated quinolone resistance, *WT* wild type

## Discussion

*S.* Typhimurium is one of the most prevalent *Salmonella* serotypes in the world, which was frequently resistant to ampicillin, amoxicillin/clavulanic acid, ceftriaxone, chloramphenicol, kanamycin, nalidixic acid, streptomycin, trimethoprim-sulfamethoxazole, and tetracyclines [28–30]. In this study, the *S.* Typhimurium isolates examined exhibited high rates of resistance to older-generation antimicrobials, and MDR (91.1%) showed the predominant resistance profile over the potential 10 year in Henan province, of which, 51.0% exhibited the ACSSuT resistance pattern. CEP, CIP, and AZI are recommended as first-line treatments for *Salmonella* infections [5, 6], and surprisingly, resistance rates to these antibiotics among the isolates tested were far higher than the values given in the 2014 National Antimicrobial Resistance Monitoring System (NARMS) report [27]. In addition, we found that the resistance rate of CEP and AZI showed a rise over time, while the resistance rate of CIP began to decline. Of concern, 91.1% of the isolates in the present investigation were MDR, an estimate higher than those in previous studies in Guangdong and Shanghai and in the abovementioned report [27, 31, 32]. More importantly, it is noteworthy that 13 (8.84%) isolates were co-resistant to CIP and CEP, and that all of these were also resistant to at least five additional antimicrobial classes, indicating that CIP and CEP resistant *S*. Typhimurium isolates have been disseminated among communities in Henan. Notably, three of the 13 isolates also exhibited resistance to AZI. Only some clinical *S.* Typhimurium strains have previously been documented with this resistance profile in China, 12 (2%) of 546 *S*. Typhimurium isolates resistant to both CIP and AXO were recovered from patients in hospitals during the period of 2005 to 2011; among these 12 isolates, two were also resistant to AZI [13]. And ACSSuT *Salmonella* co-resistant to quinolones and cephalosporins will make treatment even more difficult, and the spread of these isolates will pose a real threat to global public health. The above analyses therefore reveal that *S.* Typhimurium resistance patterns have obviously changed in recent years (Table 2 and Table S1), and empirical therapy should keep pace with these changes. Of concern is 6 (4.1%) strains are resistant to all antibiotic classes tested in our study, it meant we were almost impossible to find an effective treatment for infections. This situation demands regular surveillance of antimicrobial resistance and implements an efficient infection control program. We should control the injudicious use of antibiotics, and manage the antibiotics which are readily available in pharmacies without a prescription. Beacause antibiotic resistance could be driven by antibiotic consumption, the changing composition of consumption may also reflect alterations in patterns of resistance [33]. Otherwise, antimicrobial resistance will become more and more serious with such a sustained selective pressure. Henan Province is located in Central China, representing approximately 7% of the Chinese population, and is a major transport hub with substantial population mobility. If these MDR strains identified here become as prevalent as definitive phage type 104 worldwide, a more serious threat to national and international public health would be posed. It is therefore essential that antimicrobial resistance be monitored and appropriate drugs be chosen for *S.* Typhimurium infections.

In order to facilitate understanding of the fundamental factors underlying bacterial antimicrobial resistance and establish measures for its prevention, examination of the molecular mechanisms responsible is urgently needed. *Salmonella* CIP resistance has principally been attributed to point mutations in the quinolone resistance-determining regions of genes encoding the target gyrase (*gyrA* and *gyrB*) and topoisomerase IV (*parC* and *parE*) enzymes [7, 34]. Two *gyrA* mutations affecting amino acid residues 83 and 87 have been identified in *S.* Typhimurium with high-level resistance to CIP, and variations in other target enzyme-encoding genes such as *parC* and *parE* increase such resistance [35, 36]. In the present study, 43 CIP-resistant isolates were identified, all of which had at least one *gyrA* mutation. Thirty-nine (91%) of these carried two such mutations and showed high-level resistance. One isolate exhibiting a high degree of CIP resistance carried two *gyrA* mutations (Ser83Phe and Asp87Gly), one *parC* mutation (Ser80Arg), and a single *parE* mutation (Ser458Pro). This *parE* sequence variation has been reported previously in Taiwan, Hong Kong, and Wuhan [37–39], suggesting that it has the potential to become prevalent in China. As previously described, PMQR genes such as *qnr* and *aac(6′)-Ib-cr* have also been established as conferring CIP resistance [40]. The first PMQR gene was identified in a clinical isolate of *Klebsiella pneumoniae* in 1998 [41], and to date, various PMQR gene types have been detected in clinical *Salmonella* isolates from humans worldwide [42–44]. In this study, the genes *qnrS1* and *aac(6′)-Ib-cr* were found to be present in 25.6% (11 isolates) and 16.3% (seven isolates) of the 43 CIP-resistant isolates, respectively. Interestingly, 12 isolates simultaneously carried two *gyrA* mutations, a single *parC* mutation, and at least one PMQR gene, and thus demonstrated a high level of CIP resistance. However, three isolates highly resistant to CIP harbored only single *gyrA* mutations affecting amino acid position 87. The fact that they also carried *qnrS1* or *aac(6′)-Ib-cr* indicates that the presence of PMQR genes can increase CIP MICs [37, 40].

Production of β-lactamases is considered the predominant mechanism of bacterial resistance to CEP [45]. Among the 32 CEP-resistant isolates in the current work, *bla*_OXA_ was the most frequently observed β-lactamase gene (*n* = 28), with only *bla*_OXA-1_ being detected, suggesting that it is prevalent among *S.* Typhimurium in Henan. OXA-type β-lactamases are characterized by high levels of hydrolytic activity against oxacillin and cloxacillin, and confer resistance to AMP and CEP [46], compounding the difficulty faced in choosing antimicrobials for treatment of *S.* Typhimurium infections. In recent years, the *bla*_OXA_ gene has also been recorded at high frequencies among other *Enterobacteriaceae* [47]. Previous studies have shown that CMY-2-type β-lactamases encoded by the plasmid-borne *bla*_CMY-2_ gene are the most prevalent and problematic damaging β-lactamase of such enzymes [48]. Although the *bla*_CMY-2_ resistance gene has been documented in *Salmonella* isolates from many countries [49], its presence in China has only been reported in Shandong, Shanxi, and Sichuan Provinces [50–52]. In the current study, one isolate was positive for *bla*_CMY-2_, *bla*_CTX-M_, and *bla*_OXA_ and resistant to all antimicrobial classes tested. To the best of our knowledge, this is the first report of *bla*_CMY-2_-positive *Salmonella* in Henan. Of the 32 CEP-resistant isolates in our investigation, 40% harbored *bla*_CTX-M_ genes, while 28.1% carried the *bla*_TEM-1_ resistance gene. We also found that some of these isolates harbored at least two CEP-resistance genes at the same time. As ESBL genes are usually located on antimicrobial-resistance plasmids, they can be easily transferred between different species of bacteria [53]. We should therefore pay consideration to such phenomena and make every effort to take preventive measures.

ST19 and ST34 have been shown to be common *S.* Typhimurium STs responsible for infections worldwide [54–56]. Here, these two STs were also the most commonly encountered, indicating their predominance in Henan. Of the 43 CIP-resistant isolates, 39 were categorized as ST19, indicating a relationship between this ST and resistance to CIP (*P* < 0.05). Moreover, 13 MDR isolates co-resistant to CIP and CEP were identified as ST19, suggesting that the ST19 is prevalent among MDR *S.* Typhimurium in Henan. According to our data, we note that only by combining etiological and epidemiological information can the characteristics of the spread of resistant clonal strains be better understood.

## Conclusions

We analyzed the antimicrobial resistance and basic molecular mechanisms underlying CIP and CEP resistance of *S.* Typhimurium isolates in Henan, China, and explored their genetic relatedness. These isolates not only exhibited high rates of resistance to traditional antimicrobials but also show high resistance rates to the first-line treatments for *Salmonella* infection. More importantly, we identified certain MDR isolates co-resistant to CEP, CIP, and AZI, suggesting that the choice of treatment for *Salmonella* infection has become increasingly difficult. Among the isolates, we detected various plasmid-encoded antimicrobial-resistance genes, including PMQR, ESBL, and *mphA* genes, with some isolates even carrying two or more types, posing a serious threat to global public health. Therefore, more comprehensive surveillance is essential to prevent further spread of resistant clonal strains.

## Supplementary information

**Additional file 1: Figure S1.** Trend of resistance rate of 8 uncommon antibiotics in treatment between 2006 and 2015. It was shown in the supplemental material.

**Additional file 2.** Table S1

**Additional file 3.** The original Pulsed-field gel electrophoresis (PFGE) images of clinical S. Typhimurium isolates resistant to both ciprofloxacin and cephalosporins. The PFGE profiles of the isolates were distributed in different images, and they were labelled in red below.

## Data Availability

The data sets generated and analyzed are available from the corresponding author on reasonable request.

## Abbreviations

*ESBLs*: Extended-spectrum β-lactamases
PMQR: Plasmid-mediated quinolone resistace
QRDR: Quinolone resistance determing region
*MICs*: Minimal inhibition concentration
*MLST*: Multilocus sequence typing
*gyrA*: DNA gyrase
*parC*: Topoisomerase IV

## References

[1] Majowicz SE, Musto J, Scallan E, Angulo FJ, Kirk M, O’Brien SJ, Jones TF, Fazil A, Hoekstra RM (2010). The global burden of nontyphoidal Salmonella gastroenteritis. Clin Infect Dis.

[2] Zhang J, Wei L, Kelly P, Freeman M, Jaegerson K, Gong J, Xu B, Pan Z, Xu C, Wang C (2013). Detection of Salmonella spp. using a generic and differential FRET-PCR. PloS One.

[3] Ao TT, Feasey NA, Gordon MA, Keddy KH, Angulo FJ, Crump JA (2015). Global burden of invasive Nontyphoidal Salmonella disease, 2010. Emerg Infect Dis.

[4] Ran L, Wu S, Gao Y, Zhang X, Feng Z, Wang Z, Kan B, Klena JD, Lo Fo Wong DM, Angulo FJ (2011). Laboratory-based surveillance of nontyphoidal Salmonella infections in China. Foodborne Pathog Dis.

[5] Glynn MK, Bopp C, Dewitt W, Dabney P, Mokhtar M, Angulo FJ (1998). Emergence of multidrug-resistant Salmonella enterica serotype typhimurium DT104 infections in the United States. N Engl J Med.

[6] Sjolund-Karlsson M, Joyce K, Blickenstaff K, Ball T, Haro J, Medalla FM, Fedorka-Cray P, Zhao S, Crump JA, Whichard JM (2011). Antimicrobial susceptibility to azithromycin among Salmonella enterica isolates from the United States. Antimicrob Agents Chemother.

[7] Hakanen A, Kotilainen P, Jalava J, Siitonen A, Huovinen P (1999). Detection of decreased fluoroquinolone susceptibility in salmonellas and validation of nalidixic acid screening test. J Clin Microbiol.

[8] Weill FX, Guesnier F, Guibert V, Timinouni M, Demartin M, Polomack L, Grimont PA (2006). Multidrug resistance in Salmonella enterica serotype Typhimurium from humans in France (1993 to 2003). J Clin Microbiol.

[9] Rotimi VO, Jamal W, Pal T, Sonnevend A, Dimitrov TS, Albert MJ (2008). Emergence of multidrug-resistant Salmonella spp. and isolates with reduced susceptibility to ciprofloxacin in Kuwait and the United Arab Emirates. Diagn Microbiol Infect Dis.

[10] Hohmann EL (2001). Nontyphoidal salmonellosis. Clin Infect Dis.

[11] Zhao S, Blickenstaff K, Glenn A, Ayers SL, Friedman SL, Abbott JW, PF MD (2009). Beta-lactam resistance in salmonella strains isolated from retail meats in the United States by the National Antimicrobial Resistance Monitoring System between 2002 and 2006. Appl Environ Microbiol.

[12] Vlieghe ER, Phe T, De Smet B, Veng CH, Kham C, Bertrand S, Vanhoof R, Lynen L, Peetermans WE, Jacobs JA (2012). Azithromycin and ciprofloxacin resistance in Salmonella bloodstream infections in Cambodian adults. PLoS Negl Trop Dis.

[13] Wong MH, Yan M, Chan EW, Biao K, Chen S (2014). Emergence of clinical Salmonella enterica serovar Typhimurium isolates with concurrent resistance to ciprofloxacin, ceftriaxone, and azithromycin. Antimicrob Agents Chemother.

[14] Xiao Y, Wei Z, Shen P, Ji J, Sun Z, Yu H, Zhang T, Ji P, Ni Y, Hu Z (2015). Bacterial-resistance among outpatients of county hospitals in China: significant geographic distinctions and minor differences between central cities. Microbes Infect.

[15] Xia S, Hendriksen RS, Xie Z, Huang L, Zhang J, Guo W, Xu B, Ran L, Aarestrup FM (2009). Molecular characterization and antimicrobial susceptibility of Salmonella isolates from infections in humans in Henan Province, China. J Clin Microbiol.

[16] CLSI. Performance standards for antimicrobial susceptibility testing; twenty-seven informational supplement. CLSI document M100. Wayne, PA: Clinical and Laboratory Standards Institute; 2017.

[17] Molbak K, Baggesen DL, Aarestrup FM, Ebbesen JM, Engberg J, Frydendahl K, Gerner-Smidt P, Petersen AM, Wegener HC (1999). An outbreak of multidrug-resistant, quinolone-resistant Salmonella enterica serotype typhimurium DT104. N Engl J Med.

[18] Wu F, Xu X, Xie J, Yi S, Wang J, Yang X, Yang C, Liang B, Ma Q, Li H, Song H, Qiu S (2016). Molecular characterization of Salmonella enterica Serovar Aberdeen negative for H_2_S production in China. PLoS One.

[19] Alikhan NF, Zhou Z, Sergeant MJ, Achtman M (2018). A genomic overview of the population structure of Salmonella. PLoS Genet.

[20] Spratt BG, Hanage WP, Li B, Aanensen DM, Feil EJ (2004). Displaying the relatedness among isolates of bacterial species -- the eBURST approach. FEMS Microbiol Lett..

[21] Ribot EM, Fair MA, Gautom R, Cameron DN, Hunter SB, Swaminathan B, Barrett TJ (2006). Standardization of pulsed-field gel electrophoresis protocols for the subtyping of Escherichia coli O157:H7, Salmonella, and Shigella for PulseNet. Foodborne Pathog Dis.

[22] Hunter SB, Vauterin P, Lambert-Fair MA, Van Duyne MS, Kubota K, Graves L, Wrigley D, Barrett T, Ribot E (2005). Establishment of a universal size standard strain for use with the PulseNet standardized pulsed-field gel electrophoresis protocols: converting the national databases to the new size standard. J Clin Microbiol.

[23] Kim SY, Lee SK, Park MS, Na HT (2016). Analysis of the Fluoroquinolone antibiotic resistance mechanism of Salmonella enterica isolates. J Microbiol Biotechnol.

[24] Cui X, Wang J, Yang C, Liang B, Ma Q, Yi S, Li H, Liu H, Li P, Wu Z (2015). Prevalence and antimicrobial resistance of Shigella flexneri serotype 2 variant in China. Front Microbiol.

[25] Hasman H, Mevius D, Veldman K, Olesen I, Aarestrup FM (2005). Beta-lactamases among extended-spectrum beta-lactamase (ESBL)-resistant Salmonella from poultry, poultry products and human patients in the Netherlands. J Antimicrob Chemother.

[26] Phuc Nguyen MC, Woerther PL, Bouvet M, Andremont A, Leclercq R, Canu A (2009). Escherichia coli as reservoir for macrolide resistance genes. Emerg Infect Dis.

[27] CDC. National Antimicrobial Resistance Monitoring System for Enteric Bacteria (NARMS): Human Isolates Surveillance Report for 2014 (Final Report). Atlanta, Georgia: U.S. Department of Health and Human Services, CDC, 2016.

[28] Hendriksen RS, Vieira AR, Karlsmose S, Lo Fo Wong DM, Jensen AB, Wegener HC, Aarestrup FM (2011). Global monitoring of Salmonella serovar distribution from the World Health Organization global foodborne infections network country data Bank: results of quality assured laboratories from 2001 to 2007. Foodborne Pathog Dis.

[29] CDC. Investigation Update: Multistate Outbreak of Human Salmonella Typhimurium Infections Linked to Ground Beef. 2012.

[30] Firoozeh F, Shahcheraghi F, Zahraei Salehi T, Karimi V, Aslani MM (2011). Antimicrobial resistance profile and presence of class I integrongs among Salmonella enterica serovars isolated from human clinical specimens in Tehran, Iran. Iran J Microbiol.

[31] Zhang J, Jin H, Hu J, Yuan Z, Shi W, Ran L, Zhao S, Yang X, Meng J, Xu X (2014). Serovars and antimicrobial resistance of non-typhoidal Salmonella from human patients in Shanghai, China, 2006-2010. Epidemiol Infect.

[32] Ke B, Sun J, He D, Li X, Liang Z, Ke CW (2014). Serovar distribution, antimicrobial resistance profiles, and PFGE typing of Salmonella enterica strains isolated from 2007-2012 in Guangdong, China. BMC Infect Dis.

[33] Klein EY, Van Boeckel TP, Martinez EM, Pant S, Gandra S, Levin SA, Goossens H, Laxminarayan R (2018). Global increase and geographic convergence in antibiotic consumption between 2000 and 2015. Proc Natl Acad Sci U S A.

[34] Jeong HS, Kim JA, Shin JH, Chang CL, Jeong J, Cho JH, Kim MN, Kim S, Kim YR, Lee CH (2011). Prevalence of plasmid-mediated quinolone resistance and mutations in the gyrase and topoisomerase IV genes in Salmonella isolated from 12 tertiary-care hospitals in Korea. Microbial Drug Resist (Larchmont, NY).

[35] Vila J, Ruiz J, Marco F, Barcelo A, Goni P, Giralt E, Jimenez de anta T (1994). Association between double mutation in gyrA gene of ciprofloxacin-resistant clinical isolates of Escherichia coli and MICs. Antimicrob Agents Chemother.

[36] Heisig P (1993). High-level fluoroquinolone resistance in a Salmonella typhimurium isolate due to alterations in both gyrA and gyrB genes. J Antimicrob Chemother.

[37] Ling JM, Chan EW, Lam AW, Cheng AF (2003). Mutations in topoisomerase genes of fluoroquinolone-resistant salmonellae in Hong Kong. Antimicrob Agents Chemother.

[38] Baucheron S, Chaslus-Dancla E, Cloeckaert A, Chiu CH, Butaye P (2005). High-level resistance to fluoroquinolones linked to mutations in gyrA, parC, and parE in Salmonella enterica serovar Schwarzengrund isolates from humans in Taiwan. Antimicrob Agents Chemother.

[39] Cui S, Li J, Sun Z, Hu C, Jin S, Guo Y, Ran L, Ma Y (2008). Ciprofloxacin-resistant Salmonella enterica serotype Typhimurium, China. Emerg Infect Dis.

[40] Kim JH, Cho JK, Kim KS (2013). Prevalence and characterization of plasmid-mediated quinolone resistance genes in Salmonella isolated from poultry in Korea. Avian Pathol.

[41] Martinez-Martinez L, Pascual A, Jacoby GA (1998). Quinolone resistance from a transferable plasmid. Lancet (London, Engl).

[42] Cattoir V, Weill FX, Poirel L, Fabre L, Soussy CJ, Nordmann P (2007). Prevalence of qnr genes in Salmonella in France. J Antimicrob Chemother.

[43] Cavaco LM, Hasman H, Xia S, Aarestrup FM (2009). QnrD, a novel gene conferring transferable quinolone resistance in Salmonella enterica serovar Kentucky and Bovismorbificans strains of human origin. Antimicrob Agents Chemother.

[44] Cui S, Li J, Sun Z, Hu C, Jin S, Li F, Guo Y, Ran L, Ma Y (2009). Characterization of Salmonella enterica isolates from infants and toddlers in Wuhan, China. J Antimicrob Chemother.

[45] Miriagou V, Tassios PT, Legakis NJ, Tzouvelekis LS (2004). Expanded-spectrum cephalosporin resistance in non-typhoid Salmonella. Int J Antimicrob Agents.

[46] Bush K, Jacoby GA, Medeiros AA (1995). A functional classification scheme for beta-lactamases and its correlation with molecular structure. Antimicrob Agents Chemother.

[47] Yang C, Li P, Zhang X, Ma Q, Cui X, Li H, Liu H, Wang J, Xie J, Wu F (2016). Molecular characterization and analysis of high-level multidrug-resistance of Shigella flexneri serotype 4s strains from China. Sci Rep.

[48] Allen KJ, Poppe C (2002). Occurrence and characterization of resistance to extended-spectrum cephalosporins mediated by beta-lactamase CMY-2 in Salmonella isolated from food-producing animals in Canada. Can J Vet Res.

[49] Li XZ, Mehrotra M, Ghimire S, Adewoye L (2007). Beta-lactam resistance and beta-lactamases in bacteria of animal origin. Vet Microbiol.

[50] Li R, Lai J, Wang Y, Liu S, Li Y, Liu K, Shen J, Wu C (2013). Prevalence and characterization of Salmonella species isolated from pigs, ducks and chickens in Sichuan Province, China. Int J Food Microbiol.

[51] Yang B, Qu D, Shen J, Xi M, Zhi S, Cui S, Ji B, Meng J (2010). Antimicrobial susceptibility and related genes of Salmonella serovars from retail food in Shaanxi province. Wei Sheng Wu Xue Bao.

[52] Zhang YN, Peng J, Wang Q, Pei ZF, Zhang WJ, Niu ZX (2008). Appearance of blaCMY-2 gene-positive Salmonella isolates of pig origin in China. Int J Antimicrob Agents.

[53] Arlet G, Barrett TJ, Butaye P, Cloeckaert A, Mulvey MR, White DG (2006). Salmonella resistant to extended-spectrum cephalosporins: prevalence and epidemiology. Microbes Infect.

[54] Antunes P, Mourao J, Pestana N, Peixe L (2011). Leakage of emerging clinically relevant multidrug-resistant Salmonella clones from pig farms. J Antimicrob Chemother.

[55] Cooke FJ, Brown DJ, Fookes M, Pickard D, Ivens A, Wain J, Roberts M, Kingsley RA, Thomson NR, Dougan G (2008). Characterization of the genomes of a diverse collection of Salmonella enterica serovar Typhimurium definitive phage type 104. J Bacteriol.

[56] Antunes P, Coque TM, Peixe L (2010). Emergence of an IncIgamma plasmid encoding CMY-2 ss-lactamase associated with the international ST19 OXA-30-producing ss-lactamase Salmonella Typhimurium multidrug-resistant clone. J Antimicrob Chemother.

