# Supplementary figures and images for: The phenotypic and molecular characteristics of antimicrobial resistance of Salmonella enterica subsp. enterica serovar Typhimurium in Henan Province, China
Source: BMC Infect Dis. 2020 Jul 15;20:511. doi: 10.1186/s12879-020-05203-3 (PMC7362628; doi:10.1186/s12879-020-05203-3)

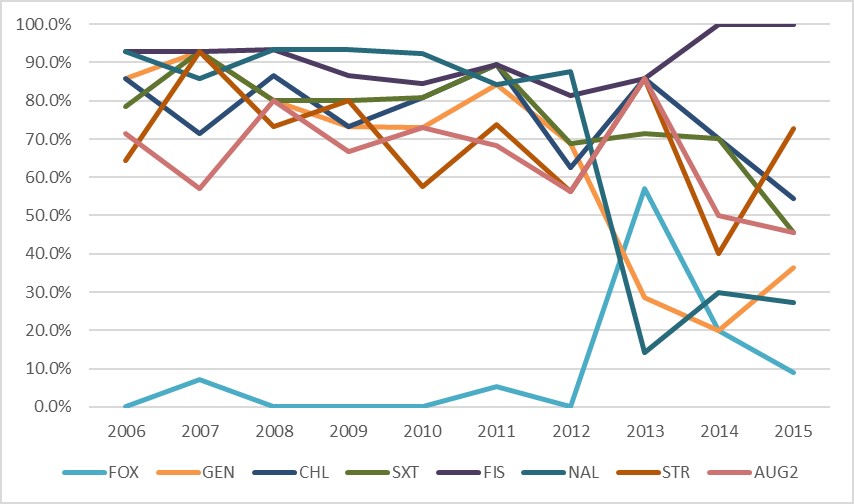

Supplement: Supplementary file 1 — Additional file 1: Figure S1. Trend of resistance rate of 8 uncommon antibiotics in treatment between 2006 and 2015. It was shown in the supplemental material. [file 12879_2020_5203_MOESM1_ESM.jpg]
