# Supplementary material for: The phenotypic and molecular characteristics of antimicrobial resistance of Salmonella enterica subsp. enterica serovar Typhimurium in Henan Province, China
Source: BMC Infect Dis. 2020 Jul 15;20:511. doi: 10.1186/s12879-020-05203-3 (PMC7362628; doi:10.1186/s12879-020-05203-3)
Supplement: Supplementary file 3 — Additional file 3. The original Pulsed-field gel electrophoresis (PFGE) images of clinical S. Typhimurium isolates resistant to both ciprofloxacin and cephalosporins. The PFGE profiles of the isolates were distributed in different images, and they were labelled in red below. [file 12879_2020_5203_MOESM3_ESM.docx]

**figure legends**

The original Pulsed-field gel electrophoresis (PFGE) images of clinical *S.* Typhimurium isolates resistant to both ciprofloxacin and cephalosporins. The PFGE profiles of the isolates were distributed in different images, and they were labelled in red below.

**
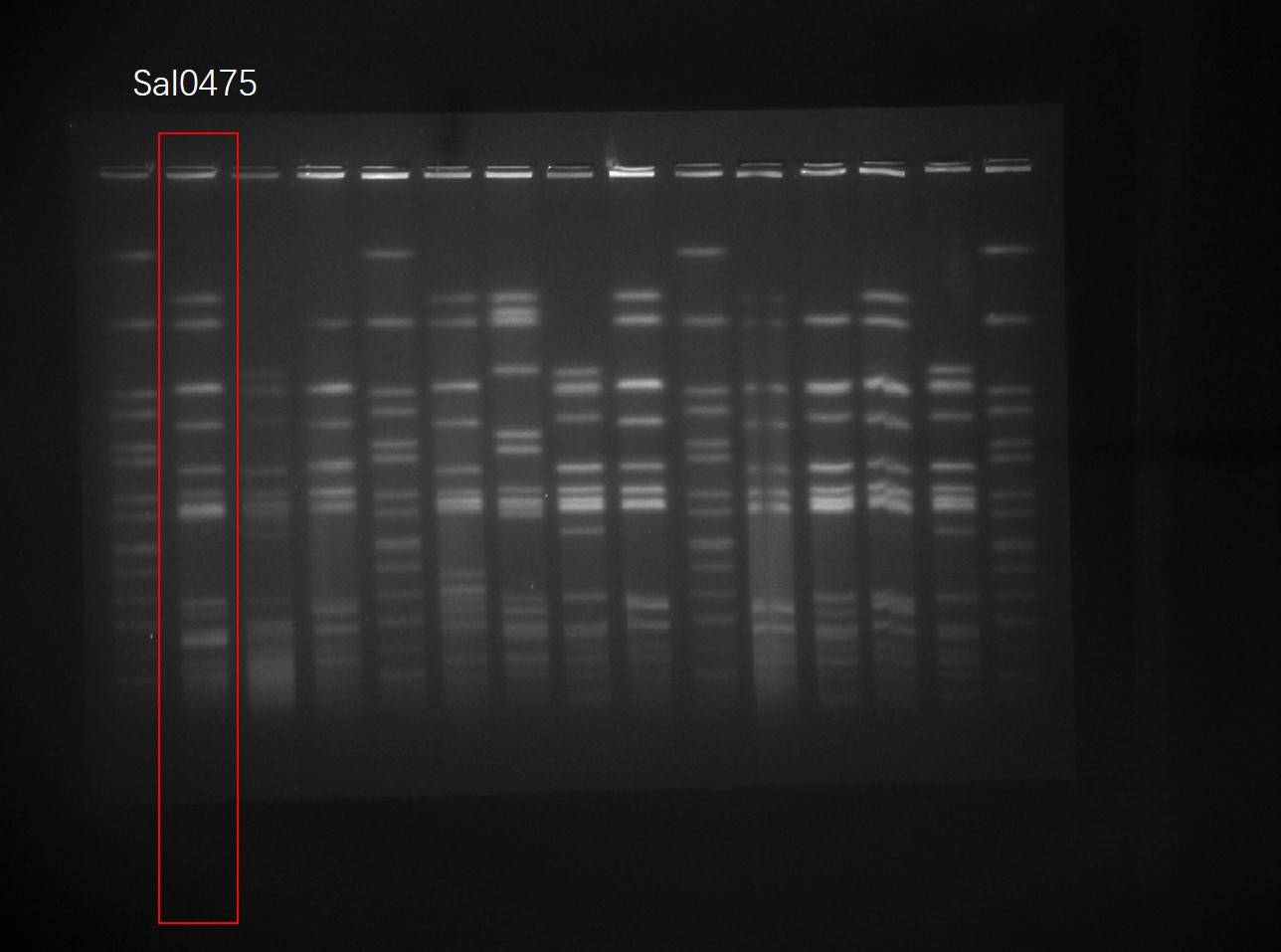

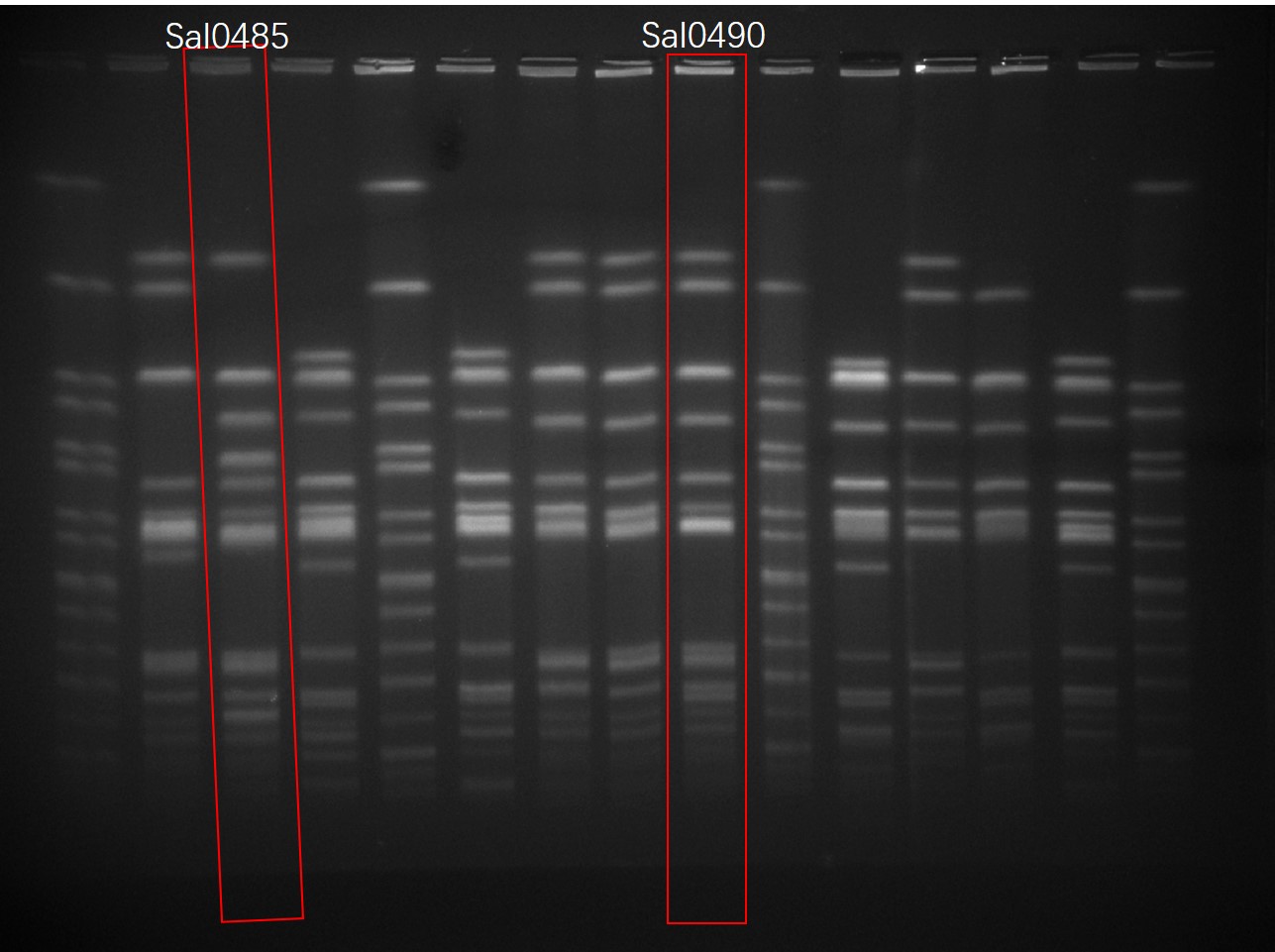

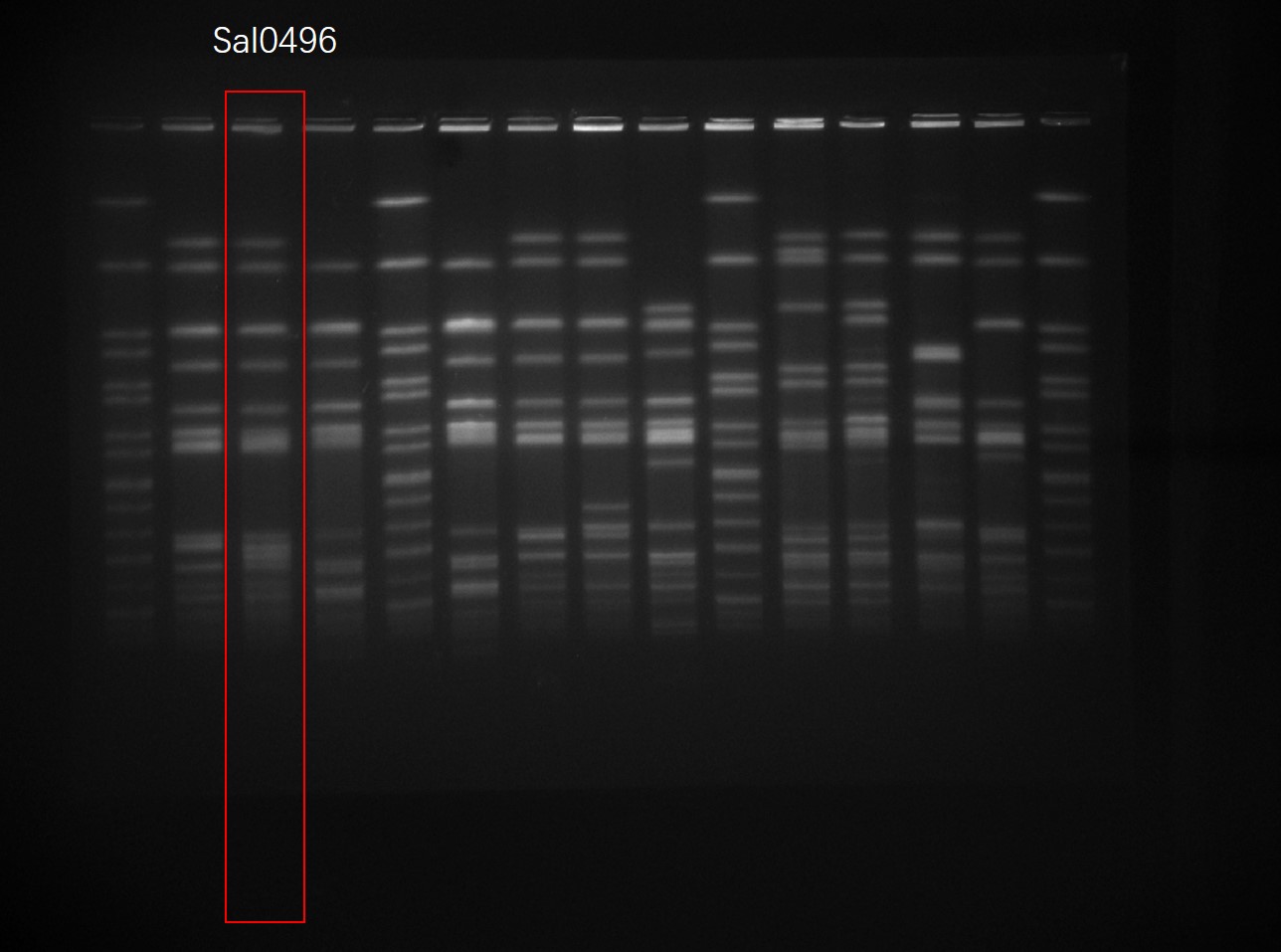

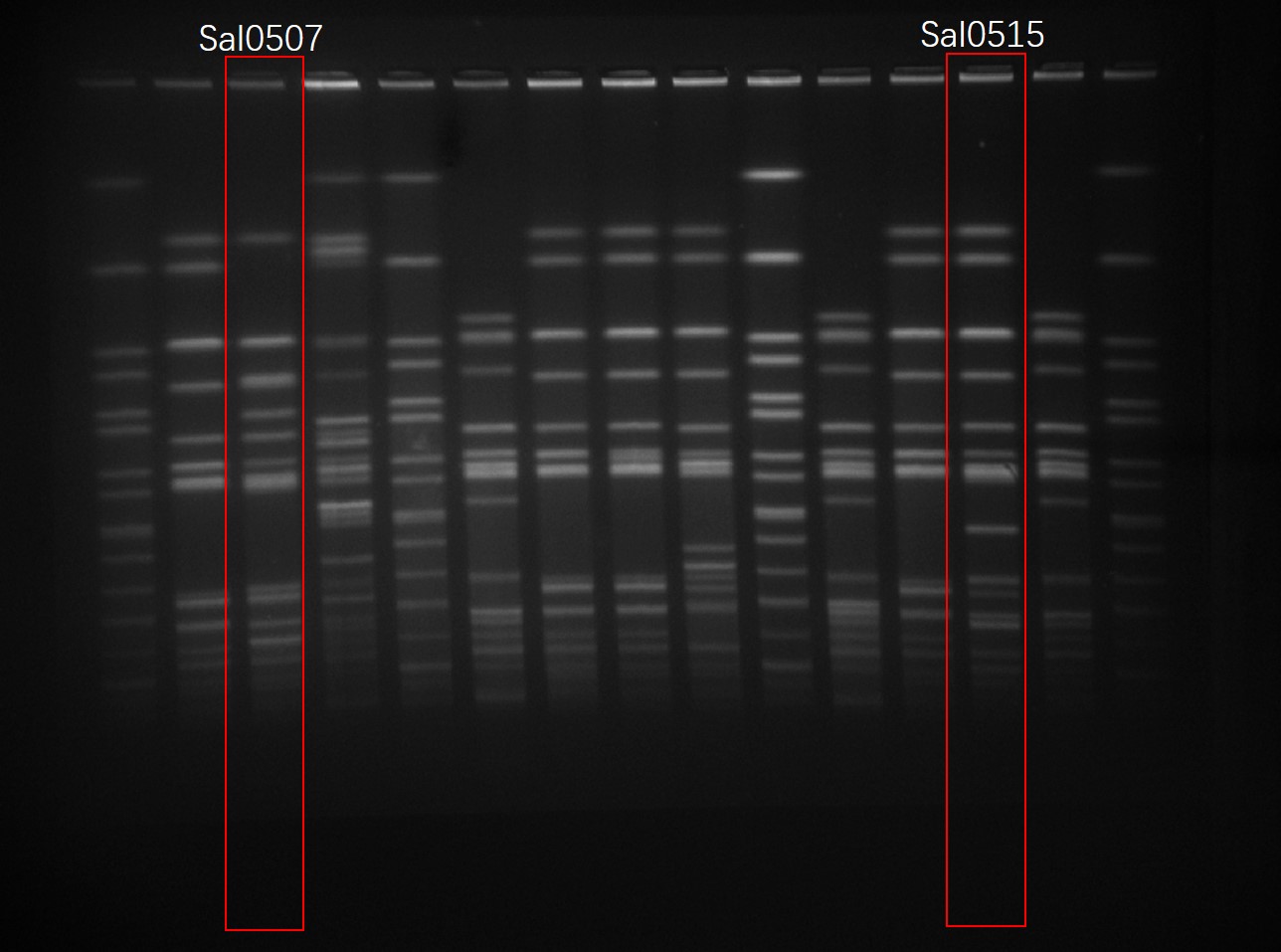

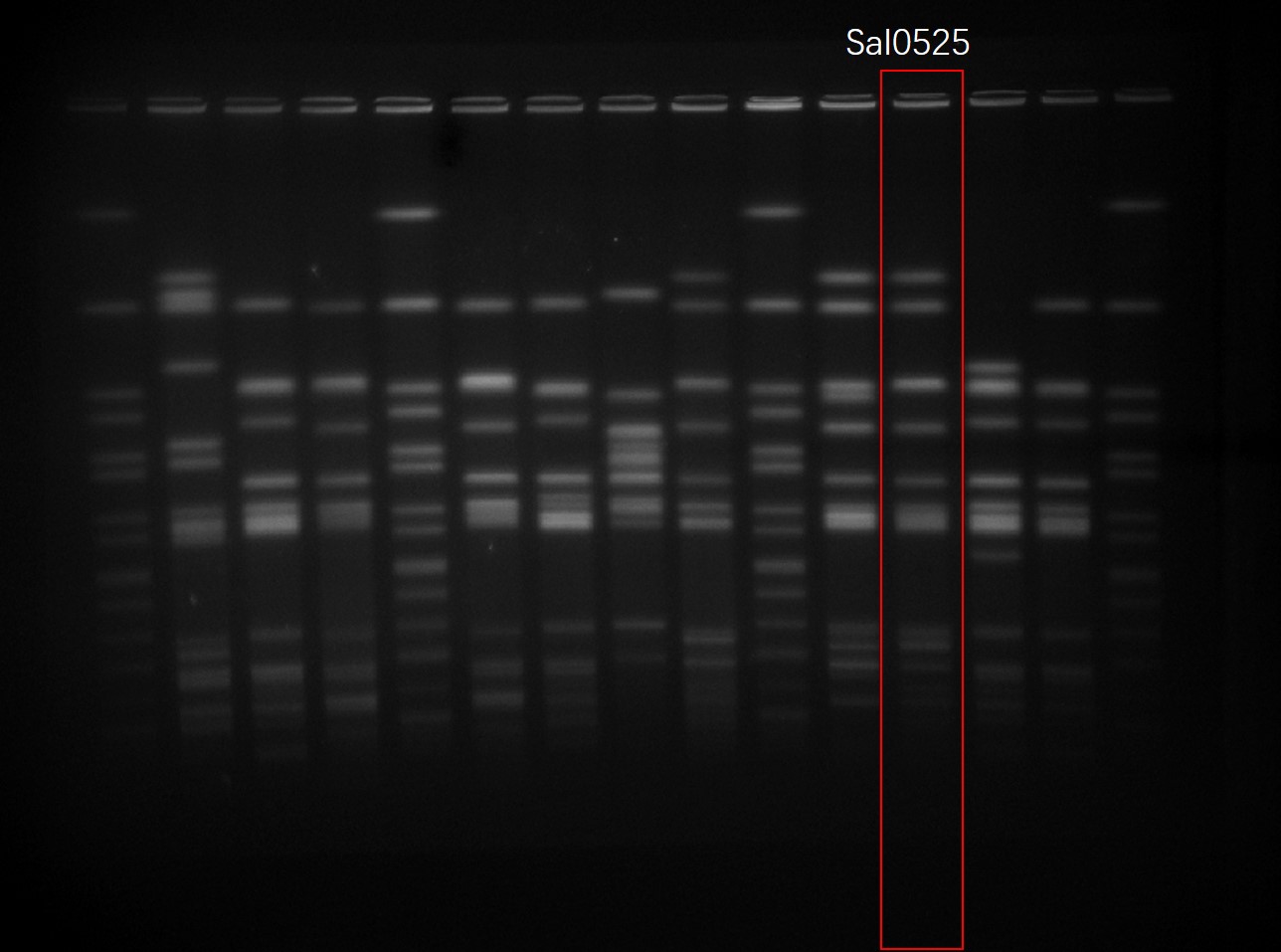

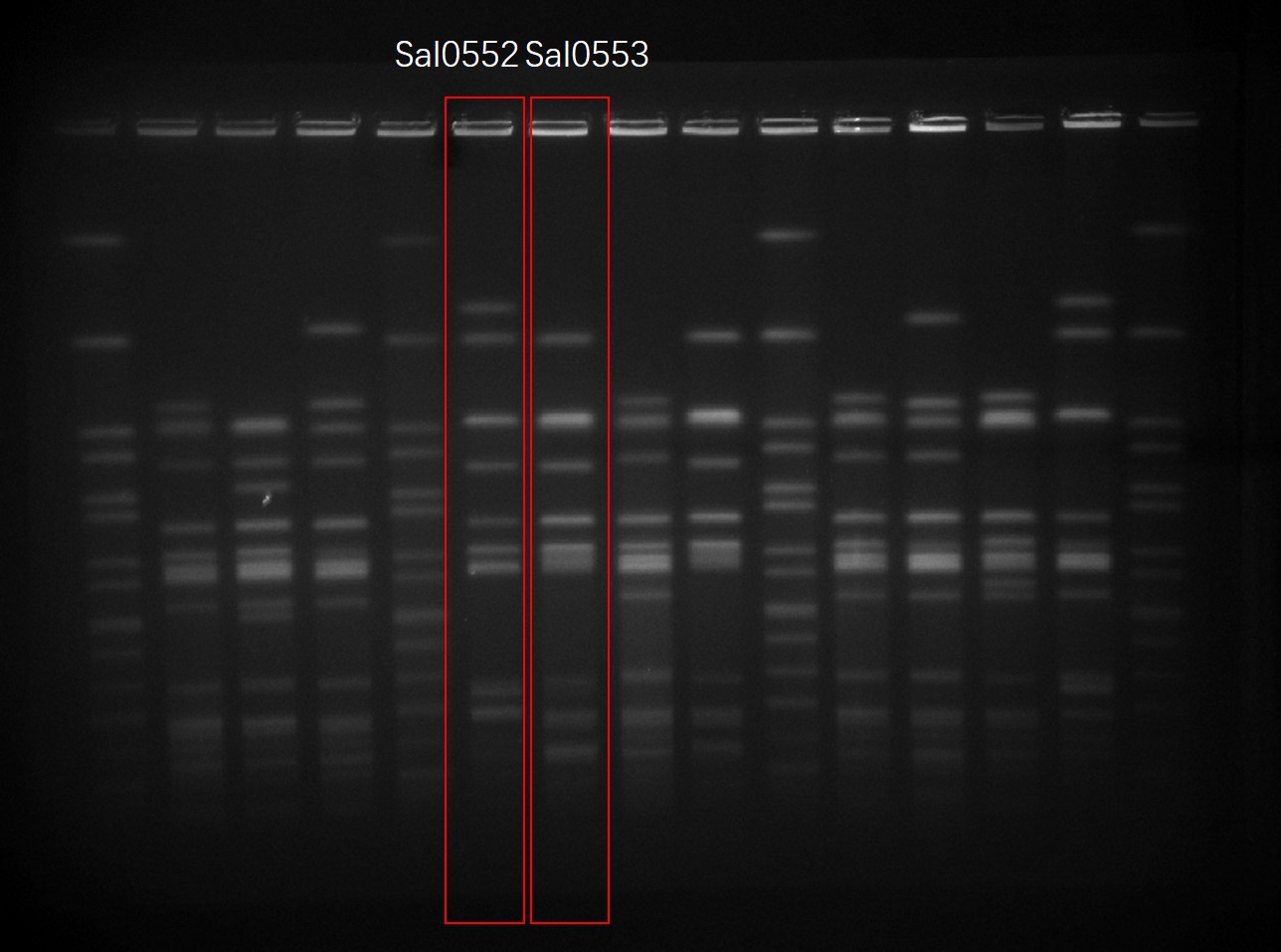

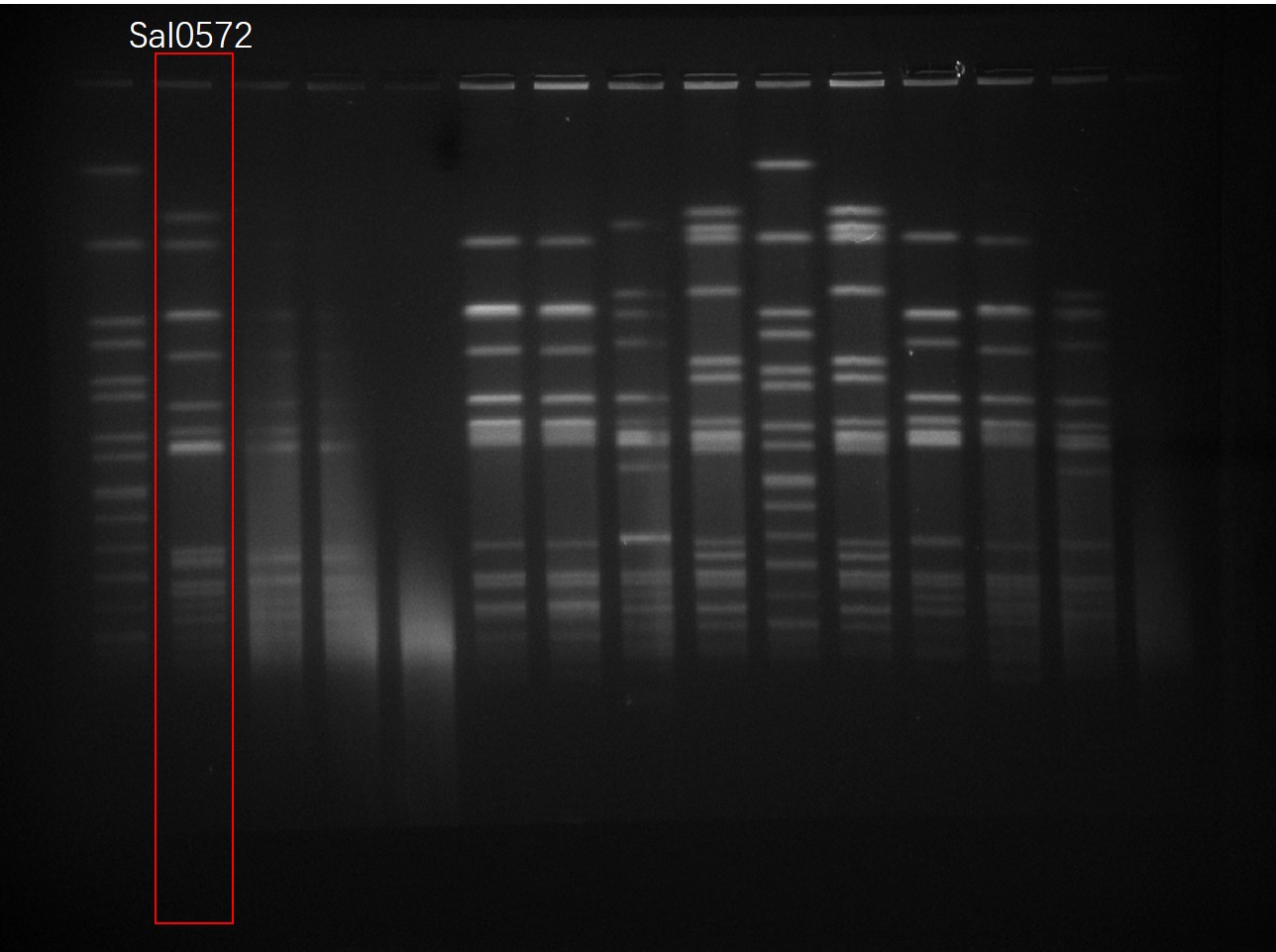

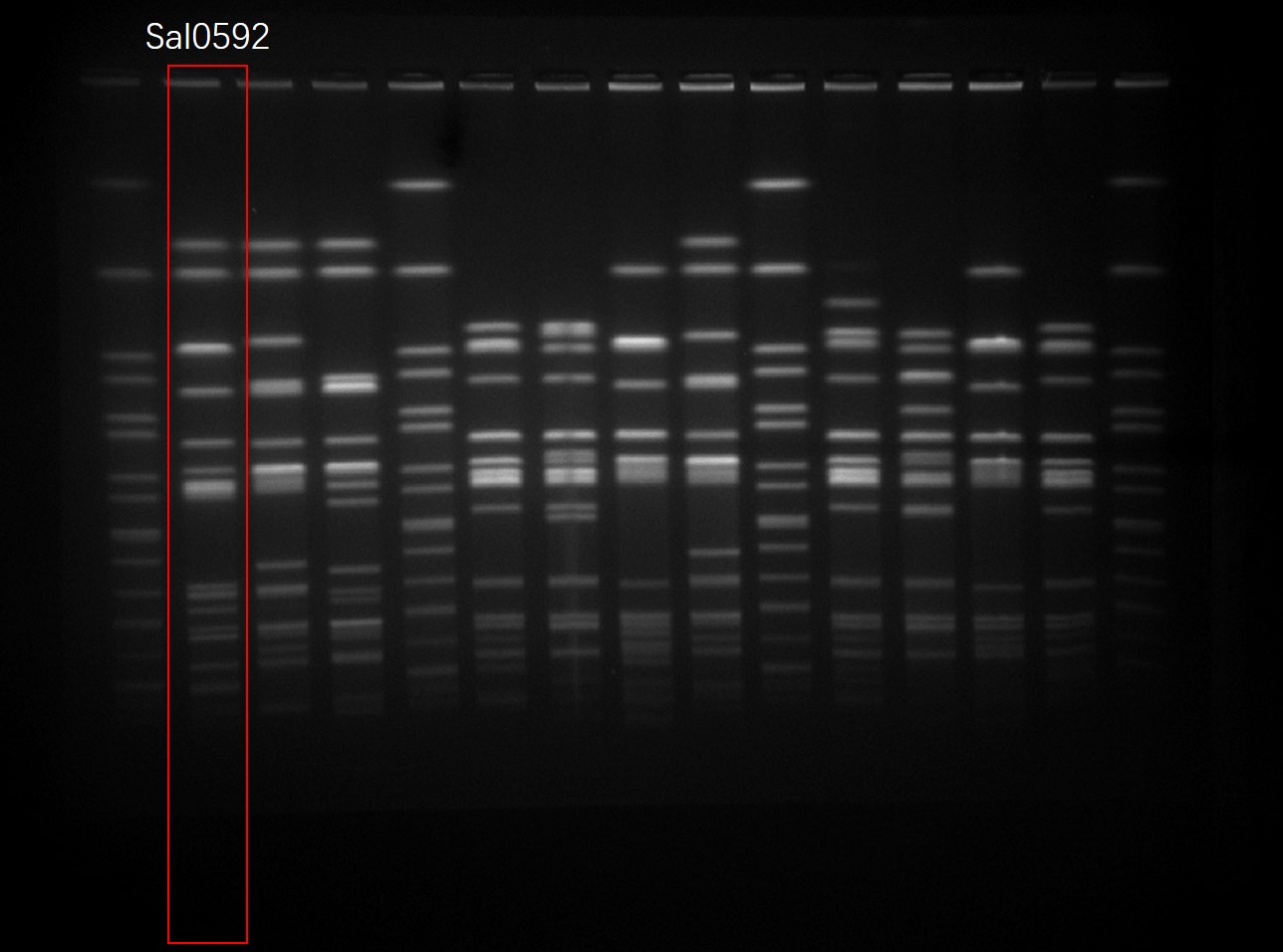

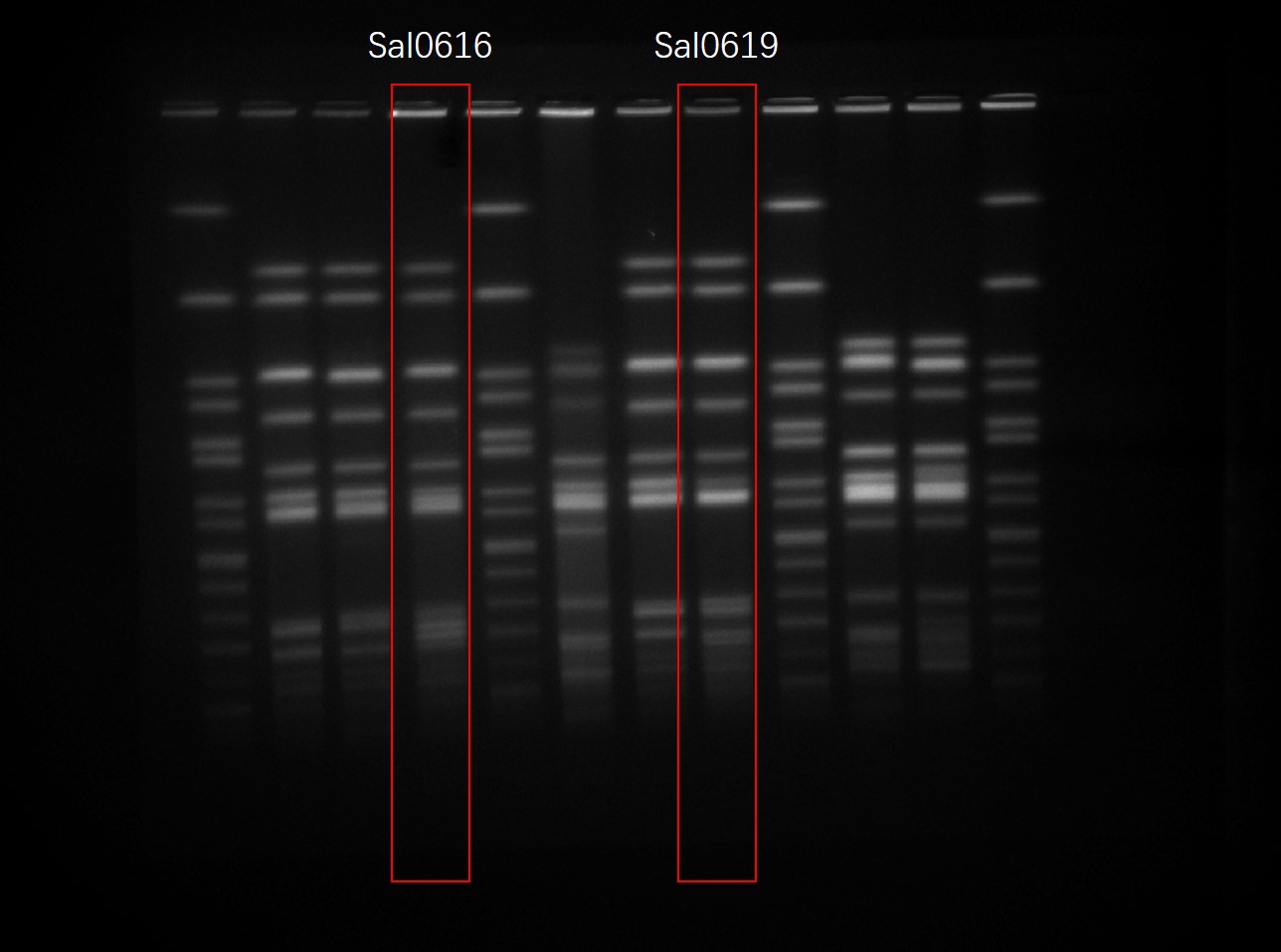
**
